# Novel Approaches for Species Concepts and Delimitation in Polyploids and Hybrids

**DOI:** 10.3390/plants11020204

**Published:** 2022-01-13

**Authors:** Elvira Hörandl

**Affiliations:** Department of Systematics, Biodiversity and Evolution of Plants (with Herbarium), University of Goettingen, 37073 Göttingen, Germany; elvira.hoerandl@biologie.uni-goettingen.de

**Keywords:** allopolyploidy, autopolyploidy, homoploid hybrids, apomixis, meiosis, speciation

## Abstract

Hybridization and polyploidization are important processes for plant evolution. However, classification of hybrid or polyploid species has been notoriously difficult because of the complexity of processes and different evolutionary scenarios that do not fit with classical species concepts. Polyploid complexes are formed via combinations of allopolyploidy, autopolyploidy and homoploid hybridization with persisting sexual reproduction, resulting in many discrete lineages that have been classified as species. Polyploid complexes with facultative apomixis result in complicated net-work like clusters, or rarely in agamospecies. Various case studies illustrate the problems that apply to traditional species concepts to hybrids and polyploids. Conceptual progress can be made if lineage formation is accepted as an inevitable consequence of meiotic sex, which is established already in the first eukaryotes as a DNA restoration tool. The turnaround of the viewpoint that sex forms species as lineages helps to overcome traditional thinking of species as “units”. Lineage formation and self-sustainability is the prerequisite for speciation and can also be applied to hybrids and polyploids. Species delimitation is aided by the improved recognition of lineages via various novel -omics methods, by understanding meiosis functions, and by recognizing functional phenotypes by considering morphological-physiological-ecological adaptations.

## 1. Introduction

The evolution and diversification of flowering plants has been largely shaped by hybridity and polyploidy. Hybridization, the merging of previously diverged genomes, is a frequent widespread phenomenon in plants, with estimates of c. 25% hybridization events related to number of angiosperm species [1,2]. Hybridization is not equally distributed taxonomically or geographically in angiosperms [1]. Nevertheless, hybrids deserve general attention as hybridization in plants can have many different outcomes: beside sterile F1 hybrid formation, hybrids may undergo further evolution via introgression, or hybrid speciation [1,3,4,5].

Hybridization can be connected to polyploidy, i.e., the multiplication of chromosome sets in the nuclear genome, which results in a whole genome duplication (WGD) or multiplication. Traditionally, polyploidization within species has been termed autopolyploidy, while polyploidy connected to hybridity as allopolyploidy, although these are just cornerstones of a variation of chromosomal configurations and meiotic behavior with many intermediate forms between multivalent formation in autopolyploids and bivalent formation in allopolyploids [6]. The mode of polyploidization is often uncertain, especially in ancient WGDs. Angiosperms have undergone several polyploidization events in their evolutionary history, and they share one ancestral whole genome duplication; hence all flowering plants are ancient polyploids (paleopolyploids) [7,8,9,10,11]. These ancient whole genome duplications have been regarded as drivers for key innovations in plants [12]. After these ancient whole genome duplications, polyploidization events happened in many plant lineages and have left further gene duplications in their genomes [7,11,12,13,14]. The more recent polyploidization events (meso- and neopolyploids; e.g., [15,16]) are important factors for plant evolution, causing potentially saltational speciation [5,17,18]. Altogether polyploidization increases diversification of flowering plants [18].

Polyploidy increases genetic and epigenetic diversity and hence allows for a higher flexibility of genetic control and gene expression patterns [6,19,20]. The merging of two genomes in allopolyploids often results in gene expression dominance of one parent [21] or in differential homeolog expression [22,23]. These factors are generally regarded as the background for improved stress response and ecological flexibility [24]. Indeed, polyploids are globally distributed and gain higher frequencies in Northern areas and under temperature extremes than diploids [25], they are overrepresented among invasive species [26], and exhibit various patterns of niche dynamics [27,28,29].

Species are regarded as the basic units of biodiversity [30]. However, it has been notoriously difficult to recognize hybrids and polyploids in species level classifications because of the complexity of evolutionary processes connected to polyploidy and hybridity [31]. These problems also cause a continued debate about percentages of hybrid/polyploid species and their contribution to plant biodiversity [18]. I will review processes resulting eventually in speciation in Section 2. Species concepts and the applicability to hybrid and polyploid lineages will be discussed under Section 3. Traditional concepts, however, do not solve the theoretical question why species exist at all [32]. Resolving the question why sex exists [33] can answer this question and explain species formation in eukaryotes (Section 4).

Species concepts define the operational criteria for species delimitation (Section 5). It is useful to keep these two processes apart [34]. However, in polyploids we will often face contradictory applications of concepts and criteria. Many taxonomists argue for pluralistic approaches and combinations of criteria for species delimitation in plants [35,36]. Molecular data, specifically DNA data, have revolutionized our understanding of evolution and classification of organisms. In the last decade we faced the transition from analysis of single-genes or DNA regions or a few hundreds of DNA fingerprints towards genomic data with a multitude of genetic information. The magnitude of data paved the way to novel analytical approaches for recognizing lineages as the first step of delimit hybrids/polyploids. However, the higher information content of -omics data does not release us from the task to find operational criteria for species delimitation. Data on reproductive biology and physiological data might help for this aspect, specifically for polyploids and hybrids.

This review will provide an overview of evolutionary processes related to hybridization and polyploidy. Based on these premises, the applicability of current species concepts to hybrids and polyploids will be discussed. A separate section will address the question why species exist as vertically evolving lineages as consequence of eukaryotic physiology, and how hybrids and polyploids can fit into this theory. I will further review progress in methodology for species delimitation with emphasis on polyploids and hybrids. This review is not intended to give all-inclusive solutions of classifications for each specific case but should stimulate thinking and gives directions for future research. A glossary of terms is provided in Glossary.

## 2. Hybrid Speciation

### 2.1. Homoploid Hybridization

Hybrid formation on the same ploidy level as both parental species is called homoploid hybridization. This process mostly results in a single F1 offspring, or in various types of persisting F1 hybrid zones [35], where F1 hybrid individuals are continuously and repeatedly formed. A hybrid zone emerges when populations of two species overlap spatially and temporally and cross to form viable offspring. In such hybrid zones, intrinsic or extrinsic selection against hybrids may further block hybrid establishment, as demonstrated in *Senecio* [37,38,39], in *Rhododendron* [40], or in *Orchis* [41].

Early generation hybrids may have various fates. In *Populus*, genomic, chromosomal and fertility studies on *P. alba* × *P. tremula* revealed a majority of F1 hybrids and selection against early-generation recombinants [42]. In shrub willows (*Salix*), a homoploid F1 hybrid zone emerged on glacier forefields within the last 120 years, occupied different micro-niches and showed high fertility; the F2 generation, however, comprised mainly backcrosses to both parents. The lack of an apparent segregation distortion indicated by linkage analysis suggested a low resistance to interspecific gene flow, which makes a scenario of introgression more likely than a potential for hybrid speciation [43,44,45]. In *Tragopogon*, sympatric European diploid species form regularly diploid hybrids with at least partial fertility, but without any indication of hybrid speciation [46]. Similarly, species of Lousiana *Iris* form hybrid zones with highly variable fertility and complex genotype-environment interactions. Introgressive hybridization instead of hybrid speciation has been inferred [47]. In *Melastoma*, molecular data confirmed the Chinese taxon *M. affine* as a hybrid lineage between *M. sanguineum* and *M. candida,* as it was assumed from morphological intermediacy; intensive backcrossing occurs with both parents [48]. Other hybrid combinations in this genus have been reported as well [49].

Hybrid speciation, however, is rare and requires reproductive isolation from the parents to prevent backcrossing and introgression [4,50]. Homoploid hybrid speciation is constrained by a narrow window of genetic divergence of the parental species: it must be low enough to allow for some viability and fertility of the first-generation hybrids, but high enough to provide a reproductive barrier against the parents [51].

Evolution of a hybrid lineage beyond the F1 generation is hard to predict. First, Mendelian segregation of the F2 generation creates a high diversity of phenotypes and genotypes. Reproductive isolation from the parents usually requires ecological separation and/or chromosomal rearrangements [50,52]. However, also cytoplasmic-nuclear incompatibilities can establish crossing barriers between species [38]. In the classical sunflower case, transgressive segregation produced genotypes with novel gene combinations and chromosomal rearrangements compared to either parent. These genotypes adapted to novel ecological niches, facilitating speciation [53]. In *Helianthus* hybrids, selection acted both on fertility and on certain phenotypic traits of hybrid lineages [54]. However, such a scenario is by no means the rule. In *Pulmonaria*, molecular data identified a homoploid hybrid species in Switzerland that has likely evolved via chromosomal changes and dysploidy as reproductive barrier against the parents; other than in sunflowers, no ecological barriers were found [55]. In *Sempervivum*, ecogeographical displacement against the parents has been identified as major source for ongoing speciation of a hybrid zone between narrowly related species [56]. Chromosomal data or fertility data of this model system are not yet available. Ecological separation also involves physiological traits, e.g., the ability of the hybrid species *Yucca gloriosa* to switch from C3 to CAM photosynthesis upon drought stress; the genetic background behind is yet unclear [57].

Most studies on proposed hybrid speciation rely on genetic evidence of hybridization while only few studies on wild plants recognize fertility data, experimental crosses and ecological data [58,59,60]. No general rule can be set on the best circumstances for homoploid hybrid speciation, it depends on the opportunities for hybrid formation and establishment [1]. However, this situation makes it difficult to apply theoretical concepts and operational criteria (see under Section 3 and Section 4 below).

### 2.2. Polyploidy

The great majority of polyploid plants maintain meiosis and syngamy, i.e., a fully sexual reproduction cycle. This differs from animals where polyploidy is usually connected to asexuality. Animals frequently have sex chromosomes, which leads in polyploids in distorted ratios of homogametic and heterogametic sexes, and hence will be selected against. Plants do not have this problem as the great majority of species has no sex chromosomes [61].

With maintaining sexual reproduction, polyploid plants have high evolutionary dynamics. Polyploidization establishes immediately a postzygotic reproductive barrier between parental taxa and their hybrid derivatives. The different chromosome sets prevent backcrossing, as the heteroploid cross between parent and hybrid would result in offspring with odd-numbered ploidy levels and high sterility [62]. Moreover, imbalance of maternal to paternal genome contributions in the endosperm after interploidy crosses can cause embryo arrest and failure of seed development [63]. Newly formed polyploids may suffer from irregular segregation of chromosomes at meiosis [6]. Allopolyploids can more easily overcome meiotic disturbances than homoploid hybrids, as the homologous doubled chromosomes from the same parent can pair, while pairing of homeologous chromosomes (derived from different parents) can be avoided [6,64]. Allopolyploids have thus also a more regular meiosis than autopolyploids, in which multivalent formation is more common [6,64]. However, established autopolyploids can return to bivalent formation via various mechanisms [65]. Establishment of a newly formed polyploid can be hampered by scarcity or lack of mating partners of the same ploidy level, a phenomenon known as minority cytotype disadvantage [66]. This problem can be overcome via shifts to uniparental reproduction by means of self-compatibility [67] or by means of facultative apomixis [68]. These reproductive systems enable single individuals to found a new population.

Speciation via allopolyploidy can happen within 100–200 years, i.e., can be saltational. The allopolyploid species of *Tragopogon* [31], *Spartina* [69,70,71], and *Mimulus* [72] are the best documented examples. In all three genera allopolyploids originated from introduced and naturalized parental species, which might have facilitated hybridization and nascent speciation. However, in many cases the allopolyploidization event may have happened in deeper time scales, i.e., within the last 100,000 years [73], or millions of years ago (e.g., in *Glycine* [74] and in *Gossypium* [75]). Taken together, allopolyploidy provides good conditions for lineage formation and is regarded a major route for hybrid speciation in plants [4,5,31,76].

**Figure 1:**
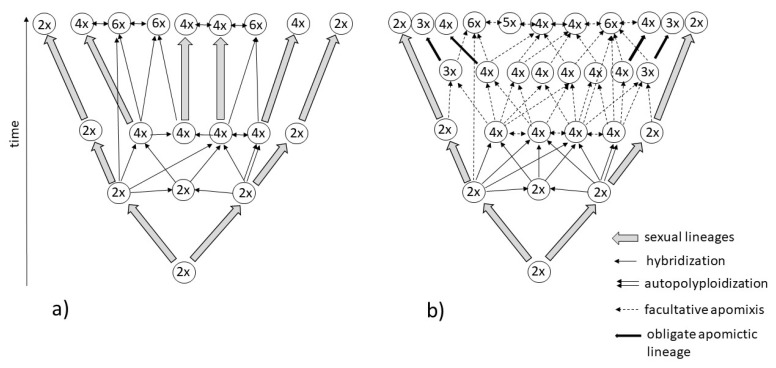
Schemes of polyploid complexes arising from two sexual progenitor species. Inter-lineage crossings between the same ploidy levels are assumed to be more successful than between ploidy levels. (**a**) Sexual polyploid complex, forming stable hybrid lineages that can be delimited as species. (**b**) Apomictic polyploid complex: sexual progenitors can be delimited as species, facultative apomicts form clusters of many hybrid biotypes; obligate apomicts could be delimited as agamospecies.

The major barrier to polyploid speciation is perhaps the rarity of successful primary polyploidization events that happen in nature mostly via unreduced gamete formation [62]. It has been further suggested that lower genetic divergence of parents results more likely in homoploid hybrid species than in allopolyploids [51,77,78]. However, these correlations observed on extant species are problematic. If the homoploid hybrid lineage goes extinct over time and only its allopolyploid derivative persists, then the allopolyploid will be clearly more diverged from its diploid parents than the original homoploid hybrid, and the parental species will be more diverged from each other than at the time of the actual origin of the hybrid (Figure 1a). Furthermore, the actual parental lineage might have experienced extinction over time, and retrospective reconstruction of parentage of an allopolyploid with extant species would reveal a more distantly related, extant species as the most likely parent. Hence, it is questionable whether genetic divergence of parental species by itself is actually a causal driver of allopolyploidization, as assumed by [77,78], or rather a by-product of post-origin divergence. Successful polyploidization rather seems to rely on conditions for unreduced gamete formation and chances for successful establishment of a polyploids.

### 2.3. Polyploid Complexes

Autopolyploidy, allopolyploidy and homoploid hybridization are by no means independent traits. They can occur together or also consecutively over larger time scales, resulting in dynamic and highly reticulate polyploid complexes, with two or more progenitor species involved in different hybrid combinations and several polyploid derivatives, with different, even multiple origins. A simple scheme for a sexual complex with two progenitors up to hexaploid cytotypes is depicted in Figure 1a.

Allopolyploids can also arise from homoploid hybrids, via unreduced gamete formation and genome doubling [76]. If the homoploid hybrid goes extinct, then reconstruction of parentage could become difficult. In mesopolyploids the parentage can be often resolved just on level of clades but not of species, e.g., in *Cardamine* from the Balcan Peninsula [79] or in *Chenopodium album* [80]. Another aspect over large time scales is the possibility of multiple origins and recurrent hybridization and polyploidization. In the Quaternary, climatic oscillations have repeatedly caused range fluctuations of species, and hence resulted in many periods of secondary contact hybridization during range expansions, and geographical isolation during range contractions [81,82,83]. These time components were often not resolved in reconstructions of evolutionary history in younger complexes that originated during the Pleistocene. Attempts to reconstruct range fluctuations via climatic data are mostly limited to the last glacial maximum (LGM), whereas the dynamics or previous range fluctuations during the Pleistocene remain hidden in darkness.

Autopolyploidization itself results more rarely in speciation [84], but can contribute to complexity of polyploid groups (Figure 1a). Autopolyploids having the same ploidy levels as related allopolyploids or other autopolyploids, make homoploid crossings among polyploid cytotypes of different origins possible. In *Veronica*, crossing barriers between species on the same tetraploid level were weaker than interploidal crosses within species [85].

In general, polyploid species cross more easily with each other than diploids do [76]. At higher ploidy levels, crossing barriers between different cytotypes become leaky as shown e.g., in *Senecio carniolicus* [86], and in *Gagea* [87]. Hybridization between related polyploid lineages appears irrespective their evolutionary origin. Hybrid populations were found e.g., in the *Cardamine pratensis* complex between autotetraploid *C. majovskyi* and polyploid cytotypes of *C. pratensis* s.str. [88]. A double hybridization event involving three parental species has given rise to allohexaploid *C. schultzii* [89]. In the *Ranunculus auricomus* complex, hybridizations of sexual autotetraploid cytotypes with sexual diploids has given rise to an allohexaploid lineage; some of these 6x populations hybridize with sympatric tetraploids and give rise to pentaploids [90,91].

The cytological background for the relaxed crossing barriers at higher ploidy levels is poorly understood; probably, the many chromosome and gene copies buffer negative effects of aneuploidy in gametes even after incorrect meiotic chromosome pairing and segregation. Another reason might be that in the great majority of angiosperms, the endosperm requires a balance of optimal ratio of two maternal (2m) and one paternal (1p) genome contributions in the endosperm because of imprinting [92,93]. Both maternal and paternal excess results in failure of viable seed formation [92]. This optimal 2m:1p ratio is more severely disturbed in interploidy crosses at lower ploidy levels than at higher ploidy levels: For instance, crosses between a diploid mother and a tetraploid father results in a 2m:2p (ratio = 1.0) in the endosperm, the reciprocal cross in a 4m:1p (ratio = 4.0). The difference to the optimal ratio is 1.0 and 2.0, respectively. Crosses between a tetraploid mother and a hexaploid father result in 4m:3p; ratio = 1.33, whereas the reciprocal cross reveals a 6m:2p endosperm, i.e., the ratio = 3.0. The difference to the optimal ratio of 2 decreases to 0.66 and 1.0, respectively. Hence, the differences to the optimal ratio of 2.0 become smaller at higher ploidy levels, and dosage effects of genomic imprinting are probably relaxed. However, little is known about the role of endosperm imbalance at higher ploidy levels. Genera or families with small or without endosperm, however, would not suffer from this problem (e.g., Asteraceae, Salicaceae, Orchidaceae, Fabaceae, Piperaceae).

At highest ploidy levels, also the distinction between allopolyploids and autopolyploids becomes blurred by transitions, as exemplified in the grass genus *Avenula* [94]. High polyploids may incorporate further genome contributions and may comprise three, four or even more parental genomes. Other cases of more than two parental species or lineages have been demonstrated in 12× to 18× *Avenula* [94], in 6× and 8× *Salix* species [95], in 6× *Chenopodium album* [80], and in 4× to 8× species of the *Ranunculus cantoniensis* complex [96]. In such cases, parentage and genome contributions can often not be reconstructed any more with certainty. In the genus *Leucanthemum*, ploidy levels range from 2× to 22×; analysis of plastid haplotypes indicate multiple origins of polyploids [97].

## 3. Species Concepts and Their Limitations for Hybrids and Polyploids

### 3.1. The Biological Species Concept (BSC)

Ernst Mayr [98] proposed the BSC as “Species are groups of actually or potentially interbreeding natural populations, which are reproductively isolated from other such groups’’. The abundance and complexity of hybridization and polyploidization in plants make a strict application of the biological species concept in practice difficult. Hence, botanists traditionally rather refused to accept the BSC, and this critical view persists up to present [31,99,100]. Interspecific hybridization happens in c. 25% of plant species and 10% of animals [2], and thus is simply too common to be neglected. Most botanists would either not regard the BSC as a primary concept for species delimitation, or they would adopt the more pragmatic approach to allow some gene exchange between species [32].

Interestingly, for the classification of plant hybrids themselves, their reproductive isolation against the parents is frequently used as one (although not exclusive) criterion, both for homoploid and allopolyploid hybrids. However, for classification of autopolyploids, the reproductive isolation between cytotypes alone is usually not seen as the sole criterion for species delimitation [84]. This inconsistency in the logics is usually not much discussed. In practice, botanists work with “fuzzy” borders of reproductive isolation and rely on case-by-case decisions.

Some authors propose complete hybrid sterility as a major criterion for a strict BSC [101]. Natural plant hybrids usually exhibit at least some fertility, which allows for production of further generations [1,100]. Hybrid sterility is hardly ever complete and highly variable [60,100]. The great variation in degree of fertility in hybrid plants makes it also impossible to use sterility of hybrids as the only operational criterion for species delimitation. Fertility of hybrids differs dramatically between different offspring classes of the same cross, as exemplified in *Iris* [1]. Selection can act upon the more fertile offspring and hence fertility of a hybrid lineage will increase over generations. Moreover, male and female development differ in their degree of fertility, even in hermaphroditic plants. Crosses between diploid sexual species of *Ranunculus* revealed in F1 and F2 hybrids much more disturbances of female meiosis and megasporogenesis than in male meiosis and microsporogenesis [102]. In hermaphrodites, these differences can be best explained by differential selection pressures on male and female development [102].

Finally, a major limitation of the BSC is the principal inapplicability to species with uniparental reproduction [32]. However, polyploid plants have a strong tendency to shift either to sexual self-compatibility or to apomixis (see Section 3.7).

### 3.2. Problems of Morphological Species Concepts in Hybrids (MSC)

Morphological species concepts are not strictly defined. A recognition of entities based on visible differences dates back historically to ancient Greek philosophers and was also followed by pre-Darwinian taxonomists [35]. Charles Darwin included an evolutionary view on species, but he presented no definition on the term species, which he regarded as sets of individuals closely resembling each other [35]. The rigorous definition of species concepts with clearly defined criteria started with Mayr’s BSC (see above). Even within the 20th century, traditional taxonomy regarded morphological similarity within a species and distinctness from other species as important criteria. Before the advent of molecular methods, morphological differences were crucial for plant taxonomy, as other features (like behavior in animals, acoustic characteristics, etc.) are not available. Early taxonomists used typological methods based on few characters and herbarium specimens [103]. The subjectivity of typological concepts is nowadays largely replaced by exact morphometric (geometric morphometric) character scoring and statistical multivariate analysis to recognize objectively phenetic differences. Still, in modern textbooks of plant taxonomy, phenetic distinction is recommended as the first step in the workflow of classification [35]. In fact, in “good” sexual plants species this approach works pretty well as interbreeding populations do exchange and inherit the genetic and epigenetic control mechanisms for an organism’s phenotype, and hence they share and inherit the same morphotype.

In hybrids, however, morphology is not a reliable predictor for a species. Hybrids are not necessarily intermediate between the parents in their phenotype and may resemble either one or the other parent, or have specific features [76]. Even F1 hybrids are not necessarily intermediate between parents, but often exhibit mismatch of characters, i.e., different characters have dominance in conflicting directions [104]. Moreover, traits segregate in the F2 and further generations in a Mendelian fashion. Crossing experiments of morphologically different diploid sexual *Ranunculus* species revealed that segregation of leaf shape in the F2 generation generates a morphospace that spans continuously the variation from one parent to another [105]. If a lineage out of such a morphospace will undergo speciation, it may resemble the one or the other parent, which may contradict the genetic relationship. For instance, phylogenomic data recognized the diploid species *R. flabellifolius* as a putative ancient, stabilized homoploid hybrid between the morphologically diverged parents *R. cassubicifolius* and *R. notabilis,* whereby its morphology resembles strongly the former species [106,107]. Morphological incongruence and mismatch likely appear in recently formed hybrids or complexes less than a Million years old. Morphology could become stabilized and species-specific in allopolyploid lineages that have persisted over longer time periods, as e.g., in polyploid willow species [95].

Finally, morphological change in polyploids/hybrids may have additionally an epigenetic background connected to the genetic one [6,108]. Studies on methylation-sensitive AFLPs suggest that epigenetic regulation alters quite significantly in polyploids/hybrid and relates to phenotypic changes [69,109,110,111,112,113]. Epigenetic changes, however, can be environmentally induced and are in plants at least partly heritable [108]. Selection can also act on different epi-genotypes even if genotypes are very similar. In perennial plants it is not trivial to discriminate between phenotypic plasticity and heritable, adaptive phenotypic change. Most hybrids and polyploids are perennials [25,114], because annuals frequently reproduce by means of self-fertilization [1,114,115].

### 3.3. Cohesion and Genotypic Cluster Concepts in Hybrids (CSC and GCSC)

Templeton’s cohesion concept [116] posits that a species is “the most inclusive population of individuals that have a potential for phenotypic cohesions though intrinsic cohesion mechanisms.” However, intrinsic cohesion is a result of gene flow, exchangeability and inheritance of the shared genetic and epigenetic background of the phenotype. This way the CSC can rely on similarity of individuals of one species. The genotypic cluster concept (GCSC) defines species as “a morphologically or genetically distinguishable group of individuals that has few or no intermediates when in contact with other such clusters” [117]. The criterion for species definition is only similarity, and some hybridization between parental species is allowed. However, such clusters can emerge only via reproductive isolation limiting gene flow with other clusters. Hence, the CSC and the GCSC represent just the reverse side of the coin of the BSC by relying on coherence within species, which results from reproductive barriers between species (although the CSC and the GCSC do not explicitly use reproductive barriers as criterion). The counter-examples of asexual polyploid complexes (see under Section 3.7) demonstrate that discrete clusters can hardly be formed without sexuality (Figure 1b).

For hybrids, J. Mallet [4] applies the same principle by regarding a hybrid species an additional, distinct multilocus genotypic cluster. For plants, clustering as a method is widely used as it can readily recognize clusters with molecular markers. But the species concept has also weaknesses. First, clustering alone does not recognize the time component, i.e., it is not known whether the cluster would persist over generations. An F1 hybrid zone could form a nice intermediate genetic cluster between the parents, but nevertheless would remain sterile and stuck in the first generation. Second, the cluster concept has no criterion for the degree of similarity to define a species. This becomes problematic in cases of sexual polyploid complexes. For instance, in the Eurasian *Achillea millefolium* complex, diploid species form nice distinct genetic clusters separated by big gaps (recognizable with AFLPs); in tetraploids, genetic clusters have no more gaps, and attach to each other or are partially overlapping, whereas in hexaploids to octoploids, clusters of pre-defined morphospecies are mostly strongly overlapping and merging [118]. The above-mentioned weakening of reproductive isolation at higher ploidy levels may explain the weakening of clustering. Third, phenotypic and genotypic clustering might be incongruent in hybrid complexes for various reasons (see above under Section 3.2).

### 3.4. Ecological Species Concepts in Hybrids and Polyploids (ESC)

The ecological species concept by Van Valen requires that “a lineage occupies an adaptive zone minimally different from that of any other lineage in its range and which evolves separately from all lineages outside its range” [119]. The ESC is tolerant to hybridization, but fails to provide a criterion for the degree of differentiation that would be required for a species. As discussed above, ecological differentiation is often used for delimitation of homoploid hybrid species, but seems less predictable for allopolyploids. Case studies using ecological niche modelling have revealed no general pattern for polyploids, but rather all types of niche expansion, contractions, shifts and stasis [27,28,120,121]. In a study on 3× to 7× cytotypes of *Allium oleraceum*, niche expansion was found in 4× to 5× cytotypes, but niche contraction occurred in high polyploids [122]. However, a study on 52 diploid-polyploid progenitor-derivative trios revealed that polyploids differentiate in their climatic niche from their progenitors more often than diploids and had higher rates of multivariate niche differentiation [29]. Ecological adaptations in polyploids often have an epigenetic component together with the genetic background [111,112,123,124], which allows a rapid response to changed environmental conditions. Polyploid lineages may acclimate and adapt rapidly—within 10 kyears—to the changed environment via a higher flexibility of gene expression and epigenetic control mechanisms, without much genetic change from diploid progenitors, e.g., in cytotypes of *Ranunculus kuepferi* [110,124]. Nevertheless, the different polyploid ecotypes are usually not classified as species if they have no other distinct features.

For integration of evolutionary lineages with ecological data time different scales come into play. Ecological situations can change very rapidly, and plants can acclimate rapidly via epigenetic modifications. Climatic fluctuations can change habitat conditions dramatically within timeframes of hundreds of years or less. Lineages, however, may exist for millions of years. In deeper time scales, information on ecology becomes rare. Some polyploid lineages have existed for millions of years, and may have undergone several niche shifts in this time period.

The strength of niche differentiation is another aspect. Theoretical models predict that disruptive selection on different habitats in sympatry must be very strong for speciation to occur, i.e., survival in the wrong niche must be very low [125]. Plants as sessile, autotrophic, photosynthetic organisms can survive in a very broad range of abiotic conditions, given the availability of water and light for photosynthesis. Hence, abiotic adaptive zones per se will be rarely exclusive within a sympatric area. The extreme case of non-survival in the wrong niche may apply to homoploid sunflower hybrids by adapting to extreme habitats like salt and sand deserts [52]. However, few such extreme ecological transitions are known in parent-hybrid derivative relationships.

Pollinator specificity is often regarded as ecological adaptation, but it is not exclusive (with few exceptions). Even with specific pollinator syndromes and effective pollinator groups, there are usually many secondary pollinators involved [126]. For a reproductive barrier between diploids and polyploids, a pollinator shift would be expected. A survey of animal-pollinated hybrids and polyploids (37 taxa) revealed pollinator shifts in c. 75% of hybridogenetic (homoploid and allopolyploid) species compared to their progenitors, while in autopolyploids it was just one case (11%) [127]. Pollinator shifts were mostly inferred from floral morphology, scent and nectar production, which follows the same principles and problems as discussed under the morphological species concept (see above). However, these proportions are not representative for hybrids and polyploids as a whole because species with generalist flowers, anemophilous and ambophilous species were not included in this study. Many plant families with frequent hybridization and polyploidy, however, have a rather uniform flower morphology (e.g., Brassicaceae), or are both wind- and animal pollinated (e.g., Salicaceae) or only wind-pollinated (e.g., Poaceae, Fagaceae, Betulaceae).

Taken together, the ESC is not universally applicable, but helpful for delimitation of younger hybrids and polyploids at shallow time scales.

### 3.5. Evolutionary Lineage Species Concepts (EvoSC): Where to Draw the Borders?

The EvoSC by E. Wiley, defines species as “a single lineage of ancestral-descendent populations or organism which maintains its identity from other such lineages and which has its own evolutionary tendencies and historical fate” [128]. Similar as with the BSC, reproductive isolation is required to maintain the lineage [32], but like in cluster concepts occasional hybridization would not invalidate a species. A major drawback is the lack of a criterion for the degree of divergence of lineages to differentiate species from infraspecific genetic structure; to put it shortly, “all species are lineages but not all lineages are species” [129]. Additional criteria are wanted. Freudenstein and coauthors [30] try to refine the concept by emphasizing that species should have a certain role in biotic interactions, depending on their phenotype (morphology, chemical compounds, behavior). The problems of phenotypic concepts in hybrids and polyploids have been discussed above (see Section 3.2).

The problem of lineage delimitation applies to diploid species as well as to polyploid and hybrid lineages. However, the EvoSC, by requesting successful reproduction over generations, helps to discriminate hybrid species from F1 hybrids and introgressive hybrid zones that got stuck in the first generations. Such early generation hybrids could form a genetic or phenetic cluster that is different from the parents, but would not form a lineage. A hybrid or autopolyploid lineage to be considered a species should have some persistence in time and space.

### 3.6. Phylogenetic Species Concepts (PSCs)

Several definitions exist for phylogenetic species concepts, the most important ones reviewed by [32]: a species could be “an irreducible (basal) cluster of organisms that is diagnosably distinct from other such clusters and within which there is a paternal pattern of ancestry and descent”; or “the smallest (exclusive) monophyletic group of common ancestry”; or “a basal, exclusive group of organisms all of whose genes coalesce more recently with each other than with those of any organisms outside the group, and that contains no exclusive group within it”. Hybridization and polyploidy fundamentally contradict principles of a phylogenetic concept (PCSs; reviewed in [32], as they neither have a single common ancestor nor do they form monophyletic groups from bifurcating splits of an ancestor into sister descendants. Both hybridization and polyploidy cause progenitor-derivative relationships, with the progenitor co-existing synchronically with its derivatives. At least initially diploids and polyploids occur also sympatrically, as a hybrid/polyploid emerges out of an outcrossing diploid population. These processes automatically create paraphyly [130,131]. Whether paraphyletic groups are acceptable in classification is much under dispute [131]. Hence PSCs are hardly ever regarded for species level taxonomy in plants. For asexual lineages, however, phylogenetic methods can be helpful to recognize shared ancestry of asexual individuals as possible criterion for recognition of ancestor-descendant lineages (see below [36]. For a PSC the problem arises that even a bifurcating phylogeny will represent a set of nested clades with no clear cut between population level, species level and genus level. There is no good criterion available at which level a clade within the nested hierarchy would be selected as a species.

### 3.7. Uniparental Reproduction and Fitness Concepts

Polyploids tend to shift to self-compatibility [67] and to apomixis [68]. Both reproductive modes allow for uniparental reproduction. Selfing is in hermaphroditic plants a frequent phenomenon, but usually remains facultative [115]. Selfing maintains meiosis separately for megasporogenesis and microsporogenesis, and hence some recombination of genetic material takes place. Hence, selfing is usually seen as a form of sexual reproduction, and the above-mentioned concepts based on sexuality can be applied. Since selfing is regarded only a short-term evolutionary strategy and associated to low diversification rates [132], there is usually not much dispute about special species concepts for selfers.

However, the evolution of polyploid complexes becomes complicated after the shift to apomixis, i.e., asexual reproduction via seeds [76]. Apomixis prevents recombination and segregation, and hence can fix a genotype over generations. However, since apomixis is hardly ever obligate, facultative sexual events can always produce new genotypes and cytotypes [68]. Aneuploid genotypes can arise more easily and maintain a clonal ancestor-descendant lineage; because of the lack of population-based clustering, these lineages are small, short-lived and numerous (Figure 1b). These processes result in numerous, heterozygous genotypes expressing slightly different morphotypes, that have been traditionally either lumped under the whole complex as one aggregate species, or each morphotype has been classified as microspecies or agamospecies [36,76,133]. Currently, four main approaches are used by taxonomists, summarized by E. Hörandl [36]: (1) classify the sexual progenitor species separately from the apomictic complex, mainly to understand better evolution and phylogenetic placement of the complex in the genus; (2) include asexual populations into the otherwise sexual progenitor species (often used for autopolyploids); (3) define bigger genetic clusters of allopolyploids as species; (4) define stable, morphologically distinct obligate asexual lineages as agamospecies. Application of this pluralistic approach often results in group specific or genus-specific classifications, and sometimes all four different approaches are realized within one complex or genus.

The zoologist B. Hausdorf [134] attempts to unify ideas of species concepts for biparentally and uniparentally reproducing taxa and defines species as “groups of individuals that are reciprocally characterized by features that would have negative fitness effects in other groups and that cannot be regularly exchanged between groups upon contact”. This differential fitness species concept differs from the BSC in that emphasis is laid on the ability of a lineage to maintain itself rather than on crossing barriers. This fits the agamospecies concept for asexual plants which also emphasizes the internal stability and persistence of lineages, and uses phenetic and ecological differences for lineage delimitation [135]. However, in obligate asexuals the criterion of negative effects of features in other groups is not practicable. If no more genetic exchange is possible between individuals or lineages due to a complete loss of sex, then it becomes also difficult to test the criterion of exchangeability as no crossing experiment could be made. One possibility of testing might be gene editing (via CRISPR-Cas9 technology) to see if the feature of another species introduced into the genome would reduce fitness.

## 4. Why Species Exist

### 4.1. How Sex Makes Species in Eukaryotes

All traditional concepts have the limitation that species are seen as given entities, and then one wonders why these entities keep apart. Here I do not intend to present a novel species concept, but rather discuss the problem of human entity-thinking. We can overcome this problem by turning around the question: why do species exist? I will discuss here how evolutionary lineages are formed in eukaryotes, and how lineage formation via sexual reproduction makes entities. Asexual lineage formation and human perception of these entities will be addressed in Section 4.2 and Section 4.3, respectively.

The question why species exist remained poorly addressed in the huge literature on speciation and species concepts. Coyne and Orr [32] based on [136] provide a survey of ideas on the observed discontinuities in nature: (1) species would exist because of self-organizing properties of biological matter; (2) species exist because they fill discrete ecological niches; (3) species exist because reproductive barriers are an inevitable result of evolutionary divergence. These ideas are not very satisfactory. First, self-organization is not a must: prokaryotes exist and evolve at a low level of self-organization (i.e., as single cells without organelles, at best forming colonies without cell and tissue differentiation). They do not follow a postulated “law” of increasing complexity [137]. The mechanisms of a postulated self-organization of biological matter remain unclear [32]. Second, the ecological niche concept reflects the thinking of ECS and the inherent problems, as outlined above. Prokaryotes can fill various ecological niches and adapt rapidly to novel environments without forming species [138]; and they can transfer genetic material from one strain to another even between strains of great genetic divergence without reproductive barriers. Species, as vertically evolving lineages, exist just in sexually reproducing eukaryotes [138]. Third, the argument of reproductive barriers as inevitable result of divergence is contradicted by rapid speciation e.g., on oceanic islands, whereas the divergence time to incompatibilities leading to sterility in interspecific crosses may take millions of years [139]. But why should reproductive barriers arise at all?

Eldredge [140], by discussing potential reasons for maintenance of sex, stated very clearly: “Species are a simple and necessary consequence of sexual reproduction”. But, this argument requires that sexual reproduction has a function on its own independent from speciation—otherwise the discussion on biological species runs into circular reasoning that species are needed to have successful sexual reproduction and sexual reproduction is good for maintaining species. An independent selective advantage is needed to explain why sexual reproduction is under positive selection. Eldregde clearly recognized the problem, but he and contemporaries failed to solve the problem as the purpose of sex was still unclear.

What is the purpose of sex? This enigmatic question is still regarded as the Queen question of evolutionary biology [141]. Many theories were proposed, but failed to provide a selective advantage of sexuality over asexuality [142]. The combinational DNA restoration theory gives an answer: meiosis evolved in ancestors of eukaryotes as a DNA repair tool of intracellular oxidative damage and DNA strand breaks [33,143,144,145,146]. In modern meiosis, chromosome pairing and double strand break formation (DSB) can scavenge DNA radicals [147]. This model is supported by observations in many eukaryotes that sex in facultative asexual organisms is triggered by increased oxidative stress [148,149,150,151,152,153,154]. Hence, this repair of oxidative lesions is a short-term, immediate advantage of sex. Subsequent repair of DSBs is in the majority of cases without genetic consequences; only the minority of DSBs results in cross-overs and recombination [155]. Incorrect repair results in chromosomal mutations that can have deleterious or beneficial consequences [155]. Reductional division at meiosis further exposes deleterious mutations in the haploid phase to purifying selection and thus avoids mutation accumulation [156,157]. This process provides a long-term advantage to sex. Merging of the two cleaned genomes of the gametes during syngamy restores diploidy, which is essential to provide two compatible chromosome sets. However, they can serve as templates for the meiotic repair functions only if their lesions are on different positions, which requires genomes of two different individuals. Predominant diploidy provides further the advantage of complementation in diplontic organisms [158], but this is not the key point of sex as also many haplontic sexual eukaryotes do exist.

According to the DNA restoration theory, meiotic sex has an indispensable, constant physiological function for preserving integrity and functionality of eukaryotic genomes from one generation to the next and in the long term. This function is highly conserved and continuously under strong, immediate positive intrinsic selection for viable gamete formation and hence for viable offspring. Most forms of asexual reproduction in eukaryotes do keep prophase I of meiosis where DNA repair takes place [159,160]. Even in putatively asexual protists, meiosis genes have been detected [161,162]. This conservation and ubiquity of meiosis underlines that sex is essential for eukaryotic physiology—in fact a consequence of this physiology, which is based on cellular oxidative respiration via mitochondria, and hence accompanied by intracellular ROS formation [145].

Meiotic sex, however, requires genome-wide compatibility of mating partners. This is not just a matter at the chromosomal level. The enzyme machinery of meiosis, especially mismatch-repair enzymes (MMR), rejects divergent DNA for homologous recombination. MMR enzymes inhibit in hybrids the formation of crossovers that are necessary for correct chromosome segregation [163]. Also correct DSB repair requires homology [155]. Hence also mismatching of neutral, non-coding DNA regions of pairing chromosomes could cause meiosis failure or disturbance. This way, meiotic compatibility acts faster and more direct than the classical model of Dobzhansky-Miller incompatibilities. Meiosis is the checkpoint for compatibility of mating partners. This compatibility, driven by selection for successful meiosis, DNA restoration and maintenance of genomic integrity, sets narrow limits of genomic divergence of mating partners. With inheritance of these compatible genome sets over generations lineages can form, and re-shuffling of alleles in populations creates the cohesive force that holds individuals of a lineage together. Because of shared genetic control mechanisms for the phenotype, the members of the lineage will share a similar morphology, physiology, and ecology and appear as a “unit”. Hence, sex makes species (not the other way-round).

The restoration theory further overcomes the teleological thinking that sex would be maintained to “create” genetic diversity in the offspring. Meiosis is not at all optimized to create new gene combinations, but restricts recombination [164]. At prophase I meiosis produces only a minimum of crossovers per chromosome that is necessary for proper segregation [143,165]. During prophase I of meiosis, many more DSBs are made that are later on resolved as crossovers and recombination events [165]. Therefore, DSBs and meiosis initiation are not selected for increasing recombination rates. Meiotic sex reduces genetic variation by limiting the choice of compatible mating partners. Bacterial transformation between divergent strains is much more efficient in creating new variants. Recombination is just a byproduct of meiosis and syngamy but not the reason for its maintenance [33]. Recombination provides just raw material for intrinsic variation on which selection can act, and hence lineages can differentiate. Recombination is not the reason for sex [33], but rather one factor for evolvability of eukaryotic lineages in time and space.

Speciation, i.e., the formation of new lineages, can happen via intrinsic change (endosymbiosis, mutations, chromosomal rearrangement, epimutation, drift, recombination, genome duplication) or extrinsic change (change of environment, climate or geomorphological situation). The new lineage will either adapt to these intrinsic/extrinsic changes and will be positively selected, or will go extinct by negative selection. Intrinsic and extrinsic change are not mutually exclusive and often difficult to disentangle. Crossing barriers between lineages arise as a by-product of this internal/external adaptation process. In plants, quite often a combination of prezygotic and postzygotic crossing barriers acts together, as shown e.g., in *Mimulus* [166,167]. It is also not useful to separate prezygotic and postzygotic crossing barriers as they often appear together [167]. In so far it also not really a problem whether speciation is sympatric or allopatric—the new lineage will form depending on the type of intrinsic or extrinsic change.

The major criterion to delimit a species is the ability of a lineage to survive and sustain itself, not the type of a crossing barrier. Selection acts primarily and continuously on mechanisms to keep integrity of the genome and the consequence is viability and fertility of a lineage. This selection acts not only on individuals but on interbreeding populations, and hence sexuality creates broader lineages that are also more robust to drift and can be maintained in some time and space. Hence, sexual reproduction, which is obligatory in 99% of plants and animals [168] created the great majority of lineages that we call species. Also in fungi, latest research rather suggests that sexuality was found in many taxa that were thought to be asexual [169]. At present it is unclear whether truly asexual fungi do exist [170]. The predominance of sex in eukaryotes is simply because the meiosis-syngamy cycle provides the most efficient DNA restoration mechanism. Very rarely, alternative mechanisms of DNA repair (e.g., gene conversion) as in the asexual Bdelloids can maintain genomic integrity in a lineage [171]. Under these conditions, also asexual diversification and speciation is possible [172].

Sexual hybrid and polyploid lineages do fit well into the DNA restoration theory because the physiological viability and heritability of the features in the lineage are important. Hybridization is at the beginning just an accident of sexual reproduction that does not affect the integrity of parental species if they can maintain purebred populations. In the hybrid, selection may act against F1 offspring after meiosis failure. We would not call these early generation hybrids a species even if they would form eventually distinct genetic clusters. However, in plants, these accidentally formed individuals can eventually restore meiosis and evolve into a new lineage. Polyploidization can be seen as a big mutation of the genome, and the newly formed polyploid has to adapt to it. The meiosis machinery adapts to the multiplication of chromosome sets in various ways [65,173,174]. Especially allopolyploids can preserve sexual reproduction and regular meiosis over generations, and hence can form a separate lineage after their origin. Allopolyploids have further the advantage of genomic novelty by combing two genomes, and this makes it more likely to speciate than in autopolyploids where just the same genome is doubled.

### 4.2. Does Asexuality Form Species?

Asexual lineages are still under the compatibility regime as long as they retain some components of meiosis (automixis in animals, autogamy in plants, and facultative apomixis belong to these “semi”-sexual forms; syngamy can be more easily skipped than meiosis [159,175]). Agamic polyploids with a mix of facultative sexuality and clonality cannot form species-like lineages, just big clusters of many local clones may appear with a reticulate pangenome-like structure as in prokaryotes [36]; Figure 1b. These clones are usually younger than sexual relatives, which could be due to neutral drift or to higher extinction risks [176]. As asexual lineages consist just of a single or few clones, they are small and can be lost by drift more easily. Polyploid clonal lineages may be still able to persist with asexual reproduction for some generations, as multiple gene copies buffer negative effects of deleterious mutations [177]. Even in polyploids low levels of sexuality suffice to counteract mutation accumulation [178]. In plants, elimination of negative mutations during the reduced haplontic phase is probably highly efficient because in the gametophytes the majority of genes is expressed and hence exposed to selection [178]. However, facultative sexuality and residual gene flow prevent that the lineage becomes distinct from others and stabilized, the whole complex rather persist with a rapid lineage-turnover.

Cytotypes with high ploidy levels (>4×) with obligate apomixis may buffer detrimental effects of DNA damage and mutation via multiple gene copies, and maintain a certain phenotype over longer time periods (e.g., in *Alchemilla*). Such stable asexual lineages might be classified as agamospecies if they are able to maintain their genomic integrity and physiological functions [36]. However, the long-term fate of apomictic polyploid complexes in plants is unknown—the fossil record cannot tell us about sexual or asexual reproduction, and hence age estimations of ancient asexual plants remain speculative. Eventually, apomictic plants could even revert to sexuality [179], as they retain the wild-type alleles for sexuality in the heterozygous or hemizygous conditions and can produce a certain proportion of fully sexually genotypes [180].

### 4.3. Why Humans Want to Classify Species

In the viewpoint above, species existed and evolved as lineages since the origin of meiosis and syngamy in first eukaryotes. Hence, species existed long before humans appeared on this planet and tried to describe species. Species are also documented in the fossil record. Consequently, it becomes clear that species are “real” and not just a construct of human thinking (see Coyne and Orr [32] for comprehensive discussion). However, human thinking and perception does have an influence as how to delimit species. We preferred traditionally morphological species concepts simply because we are visually orientated organisms. The typological thinking of species as given entities stems from a historical, pre-Darwinian view of the world that species are stable and were created by god [140]. Still, this thinking shapes most species concepts (see above) and is probably prevalent in human thinking [181]. We can overcome typological thinking by accepting that species as vertically evolving lineages are an inevitable consequence of eukaryotic physiology (see above).

The need for defining distinct units that we can name, recognize and use for our communication and biodiversity research has been discussed comprehensively before (e.g., [99]). In so far, the uncertainty of species definitions for the many hybrids and polyploids in plants is a nuisance. The difficulty to compare these species to “normal” sexual species causes big problems for quantitative estimates as how many species are there in angiosperms. Counting agamospecies in plants would result in up to ten times higher species numbers in certain regions, which is specifically problematic for temperate to arctic floras [182]. To overcome these problems, it is useful to go beyond pure phenetic approaches or the BSC, and think about species classifications that have a good conceptual background and at the same reflect our needs for communication and information content.

## 5. Novel Approaches for Species Delimitation

### 5.1. Recognition of Existing Lineages: Methodical Advances in the TaxonOMICS Era

For hybrids and polyploids a long-term success (i.e., beyond the F1 or F2) is essential, and hence lineage formation is essential. For recognition of naturally existing lineages, genomic data are the characters of choice. Because of the high frequencies of polyploidy in plants, determination of ploidy levels should be always the first step, and this can be done not only by classical chromosome counting, but also by bioinformatic methods using genome data [183]. A classical method for ploidy determination is provided by flow cytometry, which allows fast and cheap screenings of ploidy levels on a large number of samples by measuring genome size [184,185]. Basically, intact nuclei are isolated from leaf or seed tissue, stained with a DNA-sensitive fluorochrome, and then passed individually through a laser or UV-LED beam. The measured fluorescence is proportional to genome size [186]. Ploidy variation can be identified by comparison to a standard probe with known chromosome numbers [186]. Meanwhile several protocols are available to optimize measurements [187]. Knowledge of ploidy levels and rough estimates of genome size are important for planning and conducting further -omics research [188]. Further applications include flow cytometric seed screening, a method developed for screening reproductive pathways [189], that is also suitable for detecting polyploidization events during seed formation [190].

The next step is the recognition of lineages and their relationships. In genera with hybrids and polyploids, relationships of lineages are not tree-like, but reticulate. Inclusion of hybrids or polyploids in standard phylogenetic analyses can dramatically alter tree topologies [191]. Hence, both choice of molecular markers and of analytical tools must be suitable to reflect the hybrid/polyploid character of the respective lineage or complex. The widely used plastid markers or whole plastomes in plant phylogenies are mostly maternally inherited and hence cannot reflect reticulate relationships. Moreover, plastomes are often too conservative and would not provide sufficient resolution in phylogenetic analyses of less diverged lineages, as they occur in recently formed hybrids or neopolyploids [192]. Combinations of plastid and biparentally inherited nuclear markers, however, often reveal conflicting phylogenetic signals as a first indicator of reticulate relationships. Analyses of single copy-nuclear gene sequences and plastomes can be useful (e.g., in *Maddenia* [193]).

Nuclear genomes of flowering plants are characterized by big size and a high complexity [194]. Gene duplication, high levels of heterozygosity and a high fraction of repetitive elements make plant genome assembly a challenge [194,195,196,197,198]. These factors, and also the high costs for whole genome sequencing, lead most systematists to use various reduced-representation methods [199]. On shallow phylogenetic levels (population to species to infrageneric level), the restriction-enzyme based methods like RAD-Seq and GBS [200] became most popular. RAD-loci comprise a set of short-read loci from coding and non-coding regions and provide usually several thousands of SNPs. Given sufficient sequencing depth and coverage, alleles can be called, and hence these methods are efficient for recognition of closely related hybrid and polyploid lineages.

Phylogenetic analyses can give a first overview, but tree topologies must be tested for signals of hybridization. The Quartet sampling method [201] can detect conflicting phylogenetic signals at nodes as a consequence of hybridization. The in-depth analysis of putative parent-hybrid relationships requires usually a combination of methods (e.g., [95]). Distance based network analyses like NeighborNet can analyze clustering of samples and visualize better reticulate relationships [202]. Population genetic analysis tools like STRUCTURE [203] adapted for polyploids [204] or sNMF [205] can show admixture and genetic structure of a hybrid/polyploid complex. Bioinformatic tools like NewHybrids [206], HyDe [207] and SNiPloid [208] discriminate early generation hybrids and introgressants from established hybrid lineages [45,85,95,209]. The program package RADpainter and fineRADStructure allows to calculate haplotypes from RAD loci and calculates a co-ancestry matrix of pairwise similarity, which shows population and species structure at very fine resolution [210]. The disadvantages of restriction-enzyme based methods are locus dropout and hence, many missing data which requires many bioinformatic filtering and optimization steps. For polyploids, filtering paralogs and correct assembly of heterozygous loci can be a challenge, but estimates of heterozygosity are possible given sufficient sequencing depth and quality as well as appropriate clustering tresholds of loci [211,212]. Finally, RAD-data are usually re-usable only within a genus, but not across more divergent taxa [199].

Another popular method is sequencing a set of some hundreds of target-enriched nuclear genes using short (60–120 bp) probes [199]. Allele phasing for recognition of hybrids/allopolyploids is possible given sufficient sequencing depth and long reads, but might be limited in the resolution of closely related taxa if the variation in coding regions is too low. Additionally, parts of the plastomes as off-target reads are usually gained as a by-product. Both datasets can be used to reconstruct phylogenies (Hyb-Seq) and recognize incongruence in organellar and nuclear genomes [213]. A disadvantage of the target enrichment method is the need of an appropriate bioinformatic probe design for target capture of nuclear genes, which is in plants usually done from transcriptomes using low-copy protein coding genes [199]. Target enrichment data can be used to reconstruct gene trees. For polyploids, analytical pipelines became available to recognize reticulate relationships and multiple alleles of the nuclear genome [79,214,215,216]. Lineage recognition can be done efficiently via coalescent-based methods [78,107,129,217,218]. Transcriptomes are efficient in entangling relationships in polyploid complexes and can reveal allelic variation [74,219]. However, they are infrequently used for phylogenetic research because of the high costs per sample, the need of living materials, and the complex computational analysis [199].

### 5.2. Crossing Experiments, Fitness and Meiosis Studies

As outlined above, recognition of lineages alone does not suffice for species delimitation. Meiotic sex is a major mechanism for lineage formation, and hence meiosis behavior, mating compatibility, fertility and viability of offspring provide valuable additional criteria for delimitation of a species. Classical crossing experiments, microscopic investigation of meiosis, and fitness scorings are important for assessment of fertility and viability. Moreover, it would be important to understand better the background of the complex meiosis machinery although it is meanwhile known in much detail [165]. Key questions are: (1) Are the observed hotspots of DSB formation associated with oxidative DNA damage and stress? (2) Do divergent sequences of homologous chromosomes—even if on neutral sites—cause problems for chromosome pairing, DSB formation and chromosomal segregation? (3) What is the mutation rate after non-homologous repair of DSBs with the much more frequent non-crossover repair mechanisms? (4) To which extent does selection purge the mutational load in the haploid gametophyte phase? (5) How do polyploids overcome initial meiotic disturbances? Here research on non-model organisms, specifically on hybrids and polyploids, is highly wanted to understand better the checkpoints of meiosis for compatibility, and the ability of sexual reproduction for maintenance of genomic integrity of a lineage.

In hybrids and polyploids, meiosis studies and fitness parameters can help to separate F1 and early generation hybrids from established lineages. Polyploids and hybrids with reduced fertility and without lineage formation would not be classified as species but could be described with hybrid formulas or as nothotaxa [36,91,220]. In accordance with the International Code of Nomenclature for algae, fungi, and plants [221] (online available at https://www.iapt-taxon.org/nomen/main.php, accessed on 11 November 2021), nothotaxa can be designated formally with the multiplication sign in the binomial if at least one parental species is known. This way, many sexual hybrids and also many apomictic “morphospecies” could be kept separate from species classification. Autopolyploid cytotypes with lineage-specific features could be classified as species [84], but without such features they might be best sunk into the diploid progenitor or classified as subspecies.

### 5.3. Towards a Broadly Applicable Species Concept: Integrating Morphology, Physiology and Ecology

Once the self-sustainability of a lineage is established, the phenotype could serve as a recognizable criterion to delimit species. However, the phenotype is not just a set of morphological characters. For plants the sessile life style and the strong dependence on suitable abiotic conditions for performing photosynthesis imply that many adaptations, e.g., to climatic conditions, have a direct physiological background [222]. These adaptations might be not directly reflected in macro-morphology, but are usually more important for viability than visually apparent morphological characters. Experimental tests for viability of hybrids/polyploids (e.g., in common garden experiments or in controlled climate chamber experiments) will give insights into specific physiological features.

Polyploids exhibit in general a higher stress tolerance, especially to cold and drought stress [20]. The best-known physiological changes in polyploids are a general larger cell size, larger stomata cells, thereby increasing gas exchange rates and photosynthesis rates, and larger vascular cells increasing drought tolerance [120]. However, other abiotic factors need to be explored. For instance, polyploids appear to tolerate light stress of prolonged photoperiod better than diploids by more efficient quenching of excess light [223,224]. Salt tolerance in cotton was highest in allopolyploids, but most similar to respective progenitor lineages [225]. Increasing evidence suggests also a higher tolerance of polyploids to biotic stressors, e.g., pathogens, herbivores and predators [20]. Also, here -omics methods will provide further insights: both transcriptome-sequenced based and gene expression studies on certain organs via RNA-Seq are meanwhile a widely accessible method to understand adaptations [199]. Methods are available to disentangle homeolog expression in polyploids [22]. This way, we can recognize better how polyploid lineages can establish in their environment, and how lineages fill ecological niches.

Combining physiological traits to morphological features and to ecological niches can lead us to recognize a combined, adaptive phenotype that is probably more informative for lineages than discriminating morphological, physiological and ecological features in separate concepts.

## 6. Summary and Outlook

Species-level classifications have been notoriously difficult when applied to polyploids and hybrids. The complexity of processes in hybrid and polyploid plants results in many different evolutionary scenarios that do not fit to classical species concepts. The problem can be overcome by regarding species a as a consequence of lineage formation via sexual (and semi-sexual) reproduction. Meiotic sex is in eukaryotes always selected for its DNA restoration function, but requires compatibility of mating partners to work properly. Hence lineage formation is simply a consequence of meiotic sex. Novel lineages can form via intrinsic and/or extrinsic change, and selection keeps only those lineages that can adapt to this change. Under these auspices we can accept polyploid and hybrids as species if they form lineages (i.e., evolve beyond first generations) and if these lineages have a self-maintaining phenotype. This phenotype includes morphological, physiological and ecological features and helps to recognize species as basic units of biodiversity.

## Data Availability

Not applicable.

## Glossary

Allopolyploidy	Multiplication of chromosome sets connected to hybridization
Aneuploidy	Abnormal number of chromosomes, mostly uneven numbers
Apomixis	Reproduction via asexually formed seeds
Automixis	A form of asexual reproduction in animals, whereby meiosis is performed but two meiotic products fuse to restore diploidy
Autopolyploidy	Multiplication of chromosome sets within a species
Bivalent	Meiotic chromosome pairing of two homologous chromosomes
Dobzhansky-Muller-incompatibility	Model for evolution of genetic incompatibility arising from mutations in isolated, diverging lineages that are incompatible when merged again
homeolog (=homoeolog)	Duplicated chromosomes in an allopolyploid that are derived from the different parental species
Multivalent	Meiotic association of more than two chromosomes, resulting in ring formations or lagging chromosomes
Megasporogenesis	The formation of megaspores after female meiosis
Mesopolyploid	Polyploid of medium age (around 1–10 My old ^1^), with more or less stabilized meiosis and genome, with low or high chromosome numbers; intermediate period between neopolyploid and paleopolyploid. ^1^ age ranges differ between species (and authors)
Microsporogenesis	The formation of microspores after male meiosis
Minority cytotype disadvantage	Single or few individuals with a different cytotype than the majority in the population suffer from scarcity of homoploid mating partners
Neopolyploid	Recently formed polyploid (c. <1 My old ^1^), with meiotic and genomic instability, usually with low chromosome base number.
Nothotaxon	A hybrid taxon, formally indicated by a multiplication sign in the name
Paleopolyploid	Ancient polyploid (usually > 10 My old ^1^), with chromosomal behavior and genomic stability like a diploid; usually with high base chromosome number.
Segregation distortion	Departure from the expected gametic ratio of alleles in the progeny of a cross

## References

[1] Arnold M.L. (1997). Natural Hybridization and Evolution.

[2] Mallet J. (2005). Hybridization as an invasion of the genome. Trends Ecol. Evol..

[3] Stebbins G. (1950). Variation and Evolution in Plants.

[4] Mallet J. (2007). Hybrid speciation. Nature.

[5] Abbott R., Albach D., Ansell S., Arntzen J.W., Baird S.J.E., Bierne N., Boughman J., Brelsford A., Buerkle C.A., Buggs R. (2013). Hybridization and speciation. J. Evol. Biol..

[6] Comai L. (2005). The advantages and disadvantages of being polyploid. Nat. Rev. Genet..

[7] Van De Peer Y., Mizrachi E., Marchal K. (2017). The evolutionary significance of polyploidy. Nat. Rev. Genet..

[8] Leebens-Mack J.H., Barker M.S., Carpenter E.J., Deyholos M.K., Gitzendanner M.A., Graham S.W., One Thousand Plant Transcriptomes Initiative (2019). One thousand plant transcriptomes and the phylogenomics of green plants. Nature.

[9] Jiao Y., Wickett N.J., Ayyampalayam S., Chanderbali A.S., Landherr L., Ralph P.E., Soltis P.S. (2011). Ancestral polyploidy in seed plants and angiosperms. Nature.

[10] Li Z., McKibben M.T.W., Finch G.S., Blischak P.D., Sutherland B.L., Barker M.S. (2021). Patterns and Processes of Diploidization in Land Plants. Annu. Rev. Plant Biol..

[11] Wong G.K.-S., Soltis D.E., Leebens-Mack J., Wickett N.J., Barker M.S., Van De Peer Y., Graham S.W., Melkonian M. (2020). Sequencing and Analyzing the Transcriptomes of a Thousand Species Across the Tree of Life for Green Plants. Annu. Rev. Plant Biol..

[12] Soltis P.S., Soltis D.E. (2016). Ancient WGD events as drivers of key innovations in angiosperms. Curr. Opin. Plant Biol..

[13] Tuskan G.A., DiFazio S., Jansson S., Bohlmann J., Grigoriev I., Hellsten U., Rokhsar D. (2006). The genome of black cottonwood, *Populus trichocarpa* (Torr. & Gray). Science.

[14] Aköz G., Nordborg M. (2019). The *Aquilegia* genome reveals a hybrid origin of core eudicots. Genome Biol..

[15] Mandakova T., Joly S., Krzywinski M., Mummenhoff K., Lysak M.A. (2010). Fast Diploidization in Close Mesopolyploid Relatives of *Arabidopsis*. Plant Cell.

[16] Carman J.G. (1997). Asynchronous expression of duplicate genes in angiosperms may cause apomixis, bispory, tetraspory, and polyembryony. Biol. J. Linnean Soc..

[17] Soltis D.E., Visger C.J., Soltis P.S. (2014). The polyploidy revolution then and now: Stebbins revisited. Am. J. Bot..

[18] Landis J.B., Soltis D.E., Li Z., Marx H.E., Barker M.S., Tank D.C., Soltis P.S. (2018). Impact of whole-genome duplication events on diversification rates in angiosperms. Am. J. Bot..

[19] Chen Z.J. (2007). Genetic and epigenetic mechanisms for gene expression and phenotypic variation in plant polyploids. Annu. Rev. Plant Biol..

[20] Van de Peer Y., Ashman T.L., Soltis P.S., Soltis D.E. (2021). Polyploidy: An evolutionary and ecological force in stressful times. Plant Cell.

[21] Bottani S., Zabet N.R., Wendel J.F., Veitia R.A. (2018). Gene Expression Dominance in Allopolyploids: Hypotheses and Models. Trends Plant Sci..

[22] Boatwright J.L., McIntyre L.M., Morse A.M., Chen S., Yoo M.-J., Koh J., Soltis P.S., Soltis D.E., Barbazuk W.B. (2018). A Robust Methodology for Assessing Differential Homeolog Contributions to the Transcriptomes of Allopolyploids. Genetics.

[23] Akiyama R., Sun J., Hatakeyama M., Lischer H.E.L., Briskine R., Hay A., Shimizu Inatsugi R. (2021). Fine-scale empirical data on niche divergence and homeolog expression patterns in an allopolyploid and its diploid progenitor species. New Phytol..

[24] Otto S.P., Whitton J. (2000). Polyploid incidence and evolution. Annu. Rev. Genet..

[25] Rice A., Smarda P., Novosolov M., Drori M., Glick L., Sabath N., Mayrose I. (2019). The global biogeography of polyploid plants. Nat. Ecol. Evol..

[26] Te Beest M., Le Roux J.J., Richardson D.M., Brysting A.K., Suda J., Kubesova M., Pysek P. (2012). The more the better? The role of polyploidy in facilitating plant invasions. Ann. Bot..

[27] Glennon K.L., Ritchie M.E., Segraves K.A. (2014). Evidence for shared broad-scale climatic niches of diploid and polyploid plants. Ecol. Lett..

[28] Kirchheimer B., Wessely J., Gattringer G., Hülber K., Moser D., Schinkel C.C.F., Dullinger S. (2018). Reconstructing geographical parthenogenesis: Effects of niche differentiation and reproductive mode on Holocene range expansion of an alpine plant. Ecol. Lett..

[29] Baniaga A., Marx H., Arrigo N., Barker M., Early R. (2020). Polyploid plants have faster rates of multivariate niche differentiation than their diploid relatives. Ecol. Lett..

[30] Freudenstein J.V., Broe M.B., Folk R.A., Sinn B.T. (2017). Biodiversity and the Species Concept-Lineages are not Enough. Syst. Biol..

[31] Soltis P.S., Soltis D.E. (2009). The Role of Hybridization in Plant Speciation. Annu. Rev. Plant Biol..

[32] Coyne J.A., Orr H.A. (2004). Speciation.

[33] Hörandl E. (2009). A combinational theory for maintenance of sex. Heredity.

[34] De Queiroz K. (2007). Species concepts and species delimitation. Syst. Biol..

[35] Stuessy T.F. (2009). Plant Taxonomy: The Systematic Evaluation of Comparative Data.

[36] Hörandl E. (2018). The classification of asexual organisms: Old myths, new facts, and a novel pluralistic approach. Taxon.

[37] Abbott R., Brennan A.C. (2014). Altitudinal gradients, plant hybrid zones and evolutionary novelty. Phil. Trans. R. Soc. B.

[38] Brennan A.C., Hiscock S.J., Abbott R.J., Abbott R.J. (2014). Interspecific crossing and genetic mapping reveal intrinsic genomic incompatibility between two *Senecio* species that form a hybrid zone on Mount Etna, Sicily. Heredity.

[39] Brennan A., Bridle J., Hiscock S., Abbott R.J., Wang A.-L., Abbott R. (2009). Adaptation and selection in the *Senecio* (Asteraceae) hybrid zone on Mount Etna, Sicily. New Phytol..

[40] Milne R.I., Abbott R.J. (2008). Reproductive isolation among two interfertile *Rhododendron* species: Low frequency of post-F-1 hybrid genotypes in alpine hybrid zones. Mol. Ecol..

[41] Bersweden L., Viruel J., Schatz B., Harland J., Gargiulo R., Cowan R., Fay M. (2021). Microsatellites and petal morphology reveal new patterns of admixture in *Orchis* hybrid zones. Am. J. Bot..

[42] Christe C., Stölting K., Bresadola L., Fussi B., Heinze B., Wegmann D., Stolting K. (2016). Selection against recombinant hybrids maintains reproductive isolation in hybridizing *Populus* species despite F1 fertility and recurrent gene flow. Mol. Ecol..

[43] Gramlich S., Hörandl E. (2016). Fitness of natural willow hybrids in a pioneer mosaic hybrid zone. Ecol. Evol..

[44] Gramlich S., Sagmeister P., Dullinger S., Hadacek F., Hörandl E. (2016). Evolution in situ: Hybrid origin and establishment of willows (*Salix* L.) on alpine glacier forefields. Heredity.

[45] Gramlich S., Wagner N.D., Hörandl E. (2018). RAD-seq reveals genetic structure of the F-2-generation of natural willow hybrids (*Salix* L.) and a great potential for interspecific introgression. BMC Plant Biol..

[46] Matthews A., Emelianova K., Hatimy A.A., Chester M., Pellicer J., Ahmad K.S., Buggs R.J.A. (2015). 250 years of hybridization between two biennial herb species without speciation. Aob Plants.

[47] Arnold M.L., Ballerini E.S., Brothers A.N., Brothers A.N. (2012). Hybrid fitness, adaptation and evolutionary diversification: Lessons learned from Louisiana Irises. Heredity.

[48] Liu T., Chen Y., Chao L., Wang S., Wu W., Dai S., Zhou R. (2014). Extensive hybridization and introgression between *Melastoma candidum* and *M. sanguineum*. PLoS ONE.

[49] Dai S.P., Wu W., Zhang R.S., Liu T., Chen Y.Y., Shi S.H., Zhou R.C. (2012). Molecular evidence for hybrid origin of *Melastoma intermedium*. Biochem. Syst. Ecol..

[50] Abbott R.J., Hegarty M.J., Hiscock S.J., Brennan A.C. (2010). Homoploid hybrid speciation in action. Taxon.

[51] Buggs R.J.A., Soltis P.S., Soltis D.E. (2009). Does hybridization between divergent progenitors drive whole-genome duplication?. Mol. Ecol..

[52] Rieseberg L.H., Raymond O., Rosenthal D.M., Lai Z., Livingstone K., Nakazato T., Lexer C. (2003). Major ecological transitions in wild sunflowers facilitated by hybridization. Science.

[53] Rieseberg L.H., Willis J.H. (2007). Plant speciation. Science.

[54] Karrenberg S., Lexer C., Rieseberg L. (2007). Reconstructing the History of Selection during Homoploid Hybrid Speciation. Am. Nat..

[55] Gruenig S., Grünig S., Fischer M., Parisod C. (2021). Recent hybrid speciation at the origin of the narrow endemic *Pulmonaria helvetica*. Ann. Bot..

[56] Fabritzek A., Griebeler E., Kadereit J.W. (2021). Hybridization, ecogeographical displacement and the emergence of new lineages—A genotyping by sequencing (GBS) and ecological niche and species distribution modelling study of *Sempervivum tectorum* L. (Houseleek). J. Evol. Biol..

[57] Heyduk K., McAssey E.V., Grimwood J., Shu S., Schmutz J., McKain M.R., Leebens-Mack J. (2021). Hybridization history and repetitive element content in the genome of a homoploid hybrid, *Yucca gloriosa* (Asparagaceae). Front. Plant Sci..

[58] Hegarty M., Barker G., Brennan A., Edwards K., Abbott R., Hiscock S. (2008). Changes to gene expression associated with hybrid speciation in plants: Further insights from transcriptomic studies in Senecio. Philos. Trans. Biol. Sci..

[59] Schumer M., Rosenthal G., Andolfatto P. (2014). How common is homoploid hybrid speciation?. Evolution.

[60] Arnold M.L., Martin N.H. (2010). Hybrid fitness across time and habitats. Trends Ecol. Evol..

[61] Renner S.S. (2014). The relative and absolute frequencies of angiosperm sexual systems: Dioecy, monoecy, gynodioecy, and an updated online database. Am. J. Bot..

[62] Ramsey J., Schemske D.W. (1998). Pathways, mechanisms, and rates of polyploid formation in flowering plants. Annu. Rev. Ecol. Syst..

[63] Köhler C., Dziasek K., Del Toro-De Leon G. (2021). Postzygotic reproductive isolation established in the endosperm: Mechanisms, drivers and relevance. Phil. Trans. Roy. Soc. B-Biol. Sci..

[64] Cifuentes M., Grandont L., Moore G., Chèvre A.M., Jenczewski E. (2010). Genetic regulation of meiosis in polyploid species: New insights into an old question. New Phytol..

[65] Morgan C., Zhang H.K., Henry C.E., Franklin F.C.H., Bomblies K. (2020). Derived alleles of two axis proteins affect meiotic traits in autotetraploid *Arabidopsis arenosa*. Proc. Natl. Acad. Sci. USA.

[66] Levin D.A. (1975). Minority cytotype exclusion in local plant populations. Taxon.

[67] Mable B.K. (2004). Polyploidy and self-compatibility: Is there an association?. New Phytol..

[68] Hojsgaard D., Hörandl E. (2019). The Rise of Apomixis in Natural Plant Populations. Front. Plant Sci..

[69] Salmon A., Ainouche M., Wendel J.F., Wendel J.F. (2005). Genetic and epigenetic consequences of recent hybridization and polyploidy in *Spartina* (Poaceae). Mol. Ecol..

[70] Chelaifa H., Monnier A., Ainouche M. (2010). Transcriptomic changes following recent natural hybridization and allopolyploidy in the salt marsh species *Spartina × townsendii* and *Spartina anglica* (Poaceae). New Phytol..

[71] Ainouche M., Baumel A., Salmon A., Yannic G., Yannic G. (2004). Hybridization, polyploidy and speciation in *Spartina* (Poaceae). New Phytol..

[72] Vallejo Marín M., Buggs R.J.A., Cooley A., Puzey J.R., Puzey J. (2015). Speciation by genome duplication: Repeated origins and genomic composition of the recently formed allopolyploid species *Mimulus peregrinus*. Evolution.

[73] Mandáková T., Marhold K., Lysak M.A. (2014). The widespread crucifer species *Cardamine flexuosa* is an allotetraploid with a conserved subgenomic structure. New Phytol..

[74] Bombarely A., Coate J.E., Doyle J.J. (2014). Mining transcriptomic data to study the origins and evolution of a plant allopolyploid complex. PeerJ.

[75] Paterson A.H., Wendel J.F., Gundlach H., Guo H., Jenkins J., Jin D.C., Schmutz J. (2012). Repeated polyploidization of *Gossypium* genomes and the evolution of spinnable cotton fibres. Nature.

[76] Grant V. (1981). Plant Speciation.

[77] Paun O., Forest F., Fay M.F., Chase M.W. (2009). Hybrid speciation in angiosperms: Parental divergence drives ploidy. New Phytol..

[78] Wagner F., Ott T., Zimmer C., Reichhart V., Vogt R., Oberprieler C. (2019). ’At the crossroads towards polyploidy’: Genomic divergence and extent of homoploid hybridization are drivers for the formation of the ox-eye daisy polyploid complex (*Leucanthemum*, Compositae-Anthemideae). New Phytol..

[79] Šlenker M., Kantor A., Marhold K., Schmickl R., Mandáková T., Lysak M.A., Zozomová-Lihová J. (2021). Allele sorting as a novel approach to resolving the origin of allotetraploids using Hyb-Seq data: A case study of the Balkan Mountain endemic *Cardamine barbaraeoides*. Front. Plant Sci..

[80] Krak K., Vít P., Belyayev A., Douda J., Hreusová L., Mandák B., Mandak B. (2016). Allopolyploid origin of *Chenopodium album* s. str. (Chenopodiaceae): A molecular and cytogenetic insight. PLoS ONE.

[81] Hewitt G.M. (1996). Some genetic consequences of ice ages, and their role in divergence and speciation. Biol. J. Linnean Soc..

[82] Hewitt G.M. (2004). Genetic consequences of climatic oscillations in the Quaternary. Phil. Trans. R. Soc. Lond. B Biol. Sci..

[83] Hewitt G.M. (2011). Quaternary phylogeography: The roots of hybrid zones. Genetica.

[84] Soltis D.E., Soltis P.S., Schemske D.W., Hancock J.F., Thompson J.N., Husband B.C., Judd W.S. (2007). Autopolyploidy in angiosperms: Have we grossly underestimated the number of species?. Taxon.

[85] Buono D., Khan G., von Hagen K.B., Kosachev P.A., Mayland-Quellhorst E., Mosyakin S.L., Albach D.C. (2021). Comparative phylogeography of *Veronica spicata* and *V. longifolia* (Plantaginaceae) across Europe: Integrating hybridization and polyploidy in phylogeography. Front. Plant Sci..

[86] Sonnleitner M., Weis B., Flatscher R., García P., Suda J., Krejčíková J., Huelber K. (2013). Parental Ploidy Strongly Affects Offspring Fitness in Heteroploid Crosses among Three Cytotypes of Autopolyploid *Jacobaea carniolica* (Asteraceae). PLoS ONE.

[87] Pfeiffer T., Harter D.E.V., Formella N., Schnittler M. (2013). Reproductive isolation vs. interbreeding between *Gagea lutea* (L.) Ker Gawl. and *G. pratensis* (Pers.) Dumort. (Liliaceae) and their putative hybrids in Mecklenburg-Western Pomerania (Germany). Plant Spec. Biol..

[88] Melicharkova A., Slenker M., Zozomova-Lihova J., Skokanova K., Singliarova B., Kacmarova T., Marhold K. (2020). So Closely Related and Yet So Different: Strong Contrasts Between the Evolutionary Histories of Species of the *Cardamine pratensis* Polyploid Complex in Central Europe. Front. Plant Sci..

[89] Zozomova Lihova J., Mandáková T., Kovaříková A., Mühlhausen A., Mummenhoff K., Lysak M., Kovarik A. (2014). When fathers are instant losers: Homogenization of rDNAloci in recently formed *Cardamine schulzii* trigenomic allopolyploid. New Phytol..

[90] Paun O., Stuessy T.F., Hörandl E. (2006). The role of hybridization, polyploidization and glaciation in the origin and evolution of the apomictic *Ranunculus cassubicus* complex. New Phytol..

[91] Hörandl E., Greilhuber J., Klimova K., Paun O., Temsch E., Emadzade K., Hodalova I. (2009). Reticulate evolution and taxonomic concepts in the *Ranunculus auricomus* complex (Ranunculaceae): Insights from analysis of morphological, karyological and molecular data. Taxon.

[92] Köhler C., Scheid O.M., Erilova A. (2010). The impact of the triploid block on the origin and evolution of polyploid plants. Trends Genet..

[93] Friedman W.E., Madrid E.N., Williams J.H. (2008). Origin of the fittest and survival of the fittest: Relating female gametophyte development to endosperm genetics. Int. J. Plant Sci..

[94] Winterfeld G., Schneider J., Perner K., Röser M. (2012). Origin of Highly Polyploid Species: Different Pathways of Auto- and Allopolyploidy in 12x to 18x Species of *Avenula* (Poaceae). Int. J. Plant Sci..

[95] Wagner N.D., He L., Hörandl E. (2020). Phylogenomic relationships and evolution of polyploid *Salix* species revealed by RAD Sequencing data. Front. Plant Sci..

[96] Xu L.L., Li T.J., Liao L., Deng H.S., Han X.J. (2013). Reticulate evolution in *Ranunculus cantonensis* polyploid complex and its allied species. Plant Syst. Evol..

[97] Greiner R., Vogt R., Oberprieler C. (2012). Phylogenetic studies in the polyploid complex of the genus *Leucanthemum* Mill. (Compositae, Anthemideae) based on cpDNA sequence variation. Plant Syst. Evol..

[98] Mayr E. (1942). Systematics and the Origin of Species.

[99] Knapp S. (2008). Species concepts and floras: What are species for?. Biol. J. Linnean Soc..

[100] Stebbins G.L. (1959). The role of hybridization in evolution. Proc. Am. Phil. Soc..

[101] Barton N.H., Hewitt G.M. (1989). Adaptation, speciation and hybrid zones. Nature.

[102] Barke B.H., Karbstein K., Daubert M., Hörandl E. (2020). The relation of meiotic behaviour to hybridity, polyploidy and apomixis in the *Ranunculus auricomus* complex (Ranunculaceae). BMC Plant Biol..

[103] Linnaeus C. (1753). Species Plantarum.

[104] Thompson K., Urquhart Cronish M., Whitney K., Rieseberg L., Schluter D., Schluter D. (2021). Patterns, Predictors, and Consequences of Dominance in Hybrids. Am. Nat..

[105] Hodač L., Barke B.H., Hörandl E. (2018). Mendelian segregation of leaf phenotypes in experimental F-2 hybrids elucidates origin of morphological diversity of the apomictic *Ranunculus auricomus* complex. Taxon.

[106] Karbstein K., Tomasello S., Hodac L., Dunkel F.G., Daubert M., Hörandl E. (2020). Phylogenomics supported by geometric morphometrics reveals delimitation of sexual species within the polyploid apomictic *Ranunculus auricomus* complex (Ranunculaceae). Taxon.

[107] Tomasello S., Karbstein K., Hodač L., Paetzold C., Hörandl E. (2020). Phylogenomics unravels Quaternary vicariance and allopatric speciation patterns in temperate-montane plant species: A case study on the *Ranunculus auricomus* species complex. Mol. Ecol..

[108] Bossdorf O., Richards C.L., Pigliucci M. (2008). Epigenetics for ecologists. Ecol. Lett..

[109] Giraud D., Lima O., Rousseau-Gueutin M., Salmon A., Ainouche M. (2021). Gene and transposable element expression evolution following recent and past polyploidy events in *Spartina* (Poaceae). Front. Genet..

[110] Syngelaki E., Daubert M., Klatt S., Hörandl E. (2020). Phenotypic responses, reproduction mode and epigenetic patterns under temperature treatments in the alpine plant species *Ranunculus kuepferi* (Ranunculaceae). Biology.

[111] Paun O., Bateman R.M., Fay M.F., Hedren M., Civeyrel L., Chase M.W. (2010). Stable epigenetic effects impact adaptation in allopolyploid Orchids (*Dactylorhiza*: Orchidaceae). Mol. Biol. Evol..

[112] Verhoeven K.J.F., Jansen J.J., van Dijk P.J., Biere A. (2010). Stress-induced DNA methylation changes and their heritability in asexual dandelions. New Phytol..

[113] Osabe K., Clement J.D., Bedon F., Pettolino F.A., Ziolkowski L., Llewellyn D.J., Wilson I.W. (2014). Genetic and DNA methylation changes in cotton (*Gossypium*) genotypes and tissues. PLoS ONE.

[114] Stace C.A., Stace C.A. (1975). Introductory. Hybridization and the Flora of the British Islands.

[115] Richards J.A. (1997). Plant Breeding Systems.

[116] Templeton A.R., Otte D., Endler J.A. (1989). The meaning of species and speciation: A genetic perspective. Speciation and Its Consequences.

[117] Mallet J. (1995). A species definition for the modern synthesis. Trends Ecol. Evol..

[118] Guo Y.-P., Saukel J., Ehrendorfer F. (2008). AFLP trees versus scatterplots: Evolution and phylogeography of the polyploid complex *Achillea millefolium* agg. (Asteraceae). Taxon.

[119] Van Valen L. (1976). Ecological species, multispecies, and Oaks. Taxon.

[120] Soltis D., Visger C., Marchant D.B., Soltis P.S., Soltis P. (2016). Polyploidy: Pitfalls and paths to a paradigm. Am. J. Bot..

[121] Kirchheimer B., Schinkel C.C.F., Dellinger A.S., Klatt S., Moser D., Winkler M., Dullinger S. (2016). A matter of scale: Apparent niche differentiation of diploid and tetraploid plants may depend on extent and grain of analysis. J. Biogeogr..

[122] Duchoslav M., Jandová M., Kobrlová L., Šafářová L., Brus J., Vojtěchová K. (2020). Intricate distribution patterns of six cytotypes of *Allium oleraceum* at a continental scale: Niche expansion and innovation followed by niche contraction with increasing ploidy level. Front. Plant Sci..

[123] Verhoeven K.J.F., Verbon E.H., van Gurp T.P., Oplaat C., de Carvalho J.F., Morse A.M., McIntyre L.M. (2018). Intergenerational environmental effects: Functional signals in offspring transcriptomes and metabolomes after parental jasmonic acid treatment in apomictic dandelion. New Phytol..

[124] Syngelaki E., Schinkel C.C.F., Klatt S., Hörandl E. (2020). Effects of temperature treatments on cytosine-methylation profiles of diploid and autotetraploid plants of the alpine species *Ranunculus kuepferi* (Ranunculaceae) *Front*. Plant Sci..

[125] Barton N.H. (2010). What role does natural selection play in speciation?. Phil. Trans. R. Soc. B-Biol. Sci..

[126] Rosas-Guerrero V., Aguilar R., Marten-Rodriguez S., Ashworth L., Lopezaraiza-Mikel M., Bastida J.M., Quesada M. (2014). A quantitative review of pollination syndromes: Do floral traits predict effective pollinators?. Ecol. Lett..

[127] Rezende L., Suzigan J., Amorim F., Moraes A.P., Moraes A. (2020). Can plant hybridization and polyploidy lead to pollinator shift?. Acta Bot. Brasílica.

[128] Wiley E.O. (1978). Evolutionary species concept reconsidered. Syst. Zool..

[129] Sukumaran J., Knowles L.L. (2017). Multispecies coalescent delimits structure, not species. Proc. Natl. Acad. Sci. USA.

[130] Hörandl E. (2006). Paraphyletic versus monophyletic taxa-evolutionary versus cladistic classifications. Taxon.

[131] Hörandl E., Stuessy T.F. (2010). Paraphyletic groups as natural units of biological classification. Taxon.

[132] Goldberg E.E., Kohn J.R., Lande R., Robertson K.A., Smith S.A., Igić B. (2010). Species selection maintains self-incompatibility. Science.

[133] Majesky L., Krahulec F., Vasut R.J. (2017). How apomictic taxa are treated in current taxonomy: A review. Taxon.

[134] Hausdorf B. (2011). Progress toward a general species concept. Evolution.

[135] Hörandl E. (1998). Species concepts in agamic complexes: Applications in the *Ranunculus auricomus* complex and general perspectives. Folia Geobot..

[136] Maynard Smith J., Szathmary E. (1995). The Major Transitions in Evolution.

[137] McShea D.W., Brandon R.N. (2010). Biology’s First Law.

[138] Ku C., Nelson-Sathi S., Roettger M., Garg S., Hazkani-Covo E., Martin W.F. (2015). Endosymbiotic gene transfer from prokaryotic pangenomes: Inherited chimerism in eukaryotes. Proc. Natl. Acad. Sci. USA.

[139] Levin D. (2012). The long wait for hybrid sterility in flowering plants. New Phytol..

[140] Eldredge N. (1985). Unfinished Synthesis. Biological Hierarchies and Modern Evolutionary Thought.

[141] Otto S.P. (2009). The evolutionary enigma of sex. Am. Nat..

[142] Birdsell J.A., Wills C., Macintyre R.J., Clegg M.T. (2003). The evolutionary origin and maintenance of sexual recombination: A review of contemporary models. Evolutionary Biology.

[143] Bernstein H., Byerly H., Hopf F., Michod R.E., Michod R.E., Levin B.R. (1988). Is meiotic recombination an adaptation for repairing DNA, producing genetic variation, or both?. The Evolution of Sex.

[144] Bernstein C., Bernstein H. (1991). Aging, Sex and DNA Repair.

[145] Speijer D., Lukeš J., Eliáš M. (2015). Sex is a ubiquitous, ancient, and inherent attribute of eukaryotic life. Proc. Natl. Acad. Sci. USA.

[146] Hörandl E., Speijer D. (2018). How oxygen gave rise to eukaryotic sex. Proc. R. Soc. B-Biol. Sci..

[147] Hörandl E., Hadacek F. (2013). The oxidative damage initiation hypothesis for meiosis. Plant Repr..

[148] Klatt S., Hadacek F., Hodač L., Brinkmann G., Eilerts M., Hojsgaard D., Hörandl E. (2016). Photoperiod extension enhances sexual megaspore formation and triggers metabolic reprogramming in facultative apomictic *Ranunculus auricomus*. Front. Plant Sci..

[149] Mateo de Arias M., Gao L., Sherwood D.A., Dwivedi K., Price B.J., Jamison M., Carman J.G. (2020). Whether gametophytes are reduced or unreduced in angiosperms might be determined metabolically. Genes.

[150] Nedelcu A., Marcu O., Michod R. (2004). Sex as a response to oxidative stress: A twofold increase in cellular reactive oxygen species activates sex genes. Proc. R. Soc. Biol. Sci..

[151] Nedelcu A., Michod R. (2003). Sex as a response to oxidative stress: The effect of antioxidants on sexual induction in a facultatively sexual lineage. Proc. R. Soc. Biol. Sci..

[152] Hadany L., Otto S.P. (2009). Condition-dependent sex and the rate of adaption. Am. Nat..

[153] Bernstein C., Johns V. (1989). Sexual reproduction as a response to H_2_O_2_ damage in *Schizosaccharomyces pombe*. J. Bacteriol..

[154] Ulum F.B., Costa Castro C., Hörandl E. (2020). Ploidy-dependent effects of light stress on the mode of reproduction in the *Ranunculus auricomus* complex (Ranunculaceae). Front. Plant Sci..

[155] Schubert I. (2021). Boon and Bane of DNA Double-Strand Breaks. Int. J. Mol. Sci..

[156] Hörandl E., Bernstein C., Bernstein H. (2013). Meiosis and the paradox of sex in nature. Meiosis.

[157] Otto S.P., Gerstein A.C. (2008). The evolution of haploidy and diploidy. Curr. Biol..

[158] Archetti M. (2004). Recombination and loss of complementation: A more than two-fold cost for parthenogenesis. J. Evol. Biol..

[159] Mirzaghaderi G., Hörandl E. (2016). The evolution of meiotic sex and its alternatives. Proc. R. Soc. Biol. Sci..

[160] Brandeis M. (2018). New-age ideas about age-old sex: Separating meiosis from mating could solve a century-old conundrum. Biol. Rev..

[161] Kraus D., Chi J., Boenigk J., Beisser D., Graupner N., Dunthorn M. (2019). Putatively asexual chrysophytes have meiotic genes: Evidence from transcriptomic data. PeerJ.

[162] Ramesh M.A., Malik S.-B., Logsdon J.M. (2005). A phylogenomic inventory of meiotic genes: Evidence for sex in *Giardia* and an early eukaryotic origin of meiosis. Curr. Biol..

[163] Friedberg E.C., Wlaker G.C., Siede W., Wood R.D., Schultz R.A., Ellenberger T. (2006). DNA Repair and Mutagenesis.

[164] Wilkins A.S., Holliday R. (2009). The evolution of meiosis from mitosis. Genetics.

[165] Mercier R., Mézard C., Jenczewski E., Macaisne N., Grelon M., Mezard C. (2015). The Molecular Biology of Meiosis in Plants. Annu. Rev. Plant Biol..

[166] Ramsey J., Bradshaw H.D., Schemske D.W. (2003). Components of reproductive isolation between the monkeyflowers *Mimulus lewisii* and *M. cardinalis* (Phrymaceae). Evolution.

[167] Baack E., Melo M., Rieseberg L., Ortiz Barrientos D. (2015). The origins of reproductive isolation in plants. New Phytol..

[168] Burt A. (2000). Perspective: Sex, recombination, and the efficacy of selection—Was Weismann right?. Evolution.

[169] Dyer P.S., Kück U., Heitman J., Howlett B.J., Crous P.W., Stukenbrock E.H., James T.Y., Gow N.A.R. (2017). Sex and the imperfect fungi. The Fungal Kingdom.

[170] Hörandl E., Bast J., Brandt A., Scheu S., Bleidorn C., Cordellier M., Dunthorn M., Pontarotti P. (2020). Genome evolution of asexual organisms and the paradox of sex in eukaryotes. Evolutionary Biology: A Transdisciplinary Approach.

[171] Flot J.-F., Hespeels B., Li X., Noel B., Arkhipova I., Danchin E.G., Aury J.-M. (2013). Genomic evidence for ameiotic evolution in the bdelloid rotifer *Adineta vaga*. Nature.

[172] Fontaneto D., Barraclough T.G. (2015). Do Species Exist in Asexuals? Theory and Evidence from Bdelloid Rotifers. Integr. Comp. Biol..

[173] Bohutinska M., Handrick V., Yant L., Schmickl R., Kolar F., Bomblies K., Paajanen P. (2021). De novo mutation and rapidpProtein (co-)evolution during meiotic adaptation in *Arabidopsis arenosa*. Mol. Biol. Evol..

[174] Bomblies K., Higgins J.D., Yant L. (2015). Meiosis evolves: Adaptation to external and internal environments. New Phytol..

[175] Constable G.W.A., Kokko H. (2021). Parthenogenesis and the evolution of anisogamy. Cells.

[176] Janko K. (2014). Let us not be unfair to asexuals: Their ephemerality may be explained by neutral models without invoking any evolutionary constraints of asexuality. Evolution.

[177] Gerstein A.C., Otto S.P. (2009). Ploidy and the Causes of Genomic Evolution. J. Hered..

[178] Hodač L., Klatt S., Hojsgaard D., Sharbel T., Hörandl E. (2019). A little bit of sex prevents mutation accumulation even in apomictic polyploid plants. BMC Evol. Biol..

[179] Hörandl E., Hojsgaard D. (2012). The evolution of apomixis in angiosperms: A reappraisal. Plant Biosyst..

[180] Ozias-Akins P., van Dijk P.J. (2007). Mendelian genetics of apomixis in plants. Annu. Rev. Genet..

[181] Love A.C. (2009). Typology Reconfigured: From the Metaphysics of Essentialism to the Epistemology of Representation. Acta Biotheor..

[182] Stace C.A. (1998). Species recognition in agamosperms—The need for a pragmatic approach. Folia Geobot..

[183] Kyriakidou M., Tai H.H., Anglin N.L., Ellis D., Strömvik M.V. (2018). Current Strategies of Polyploid Plant Genome Sequence Assembly. Front. Plant Sci..

[184] Greilhuber J., Dolezel J., Lysák M.A., Bennett M.D. (2005). The origin, evolution and proposed stabilization of the terms ’genome size’ and ’C-value’ to describe nuclear DNA contents. Ann. Bot..

[185] Dolezel J., Greilhuber J., Suda J. (2007). Estimation of nuclear DNA content in plants using flow cytometry. Nat. Protoc..

[186] Kron P., Suda J., Husband B.C. (2007). Applications of flow cytometry to evolutionary and population biology. Annu. Rev. Ecol. Evol. Syst..

[187] Loureiro J., Kron P., Temsch E.M., Koutecky P., Lopes S., Castro M., Castro S. (2021). Isolation of plant nuclei for estimation of nuclear DNA content: Overview and best practices. Cytom. Part A.

[188] Dolezel J., Cizkova J., Simkova H., Bartos J. (2018). One Major Challenge of Sequencing Large Plant Genomes Is to Know How Big They Really Are. Int. J. Mol. Sci..

[189] Matzk F., Meister A., Schubert I. (2000). An efficient screen for reproductive pathways using mature seeds of monocots and dicots. Plant J..

[190] Schinkel C.C.F., Kirchheimer B., Dullinger S., Geelen D., De Storme N., Hörandl E. (2017). Pathways to polyploidy: Indications of a female triploid bridge in the alpine species *Ranunculus kuepferi* (Ranunculaceae). Plant Syst. Evol..

[191] McDade L.A. (1992). HYBRIDS AND PHYLOGENETIC SYSTEMATICS.1. THE IMPACT OF HYBRIDS ON CLADISTIC-ANALYSIS. Evolution.

[192] Wagner N.D., Volf M., Hörandl E. (2021). Highly diverse shrub willows (*Salix* L.) share highly similar plastomes. Front. Plant Sci..

[193] Su N., Liu B., Wang J., Tong R., Ren C., Chang Z., Wen J. (2021). On the species delimitation of the *Maddenia* Group of *Prunus* (Rosaceae): Evidence from plastome and nuclear Sequences and morphology. Front. Plant Sci..

[194] Michael T.P., VanBuren R. (2020). Building near-complete plant genomes. Curr. Opin. Plant Biol..

[195] Paajanen P., Kettleborough G., Lopez-Girona E., Giolai M., Heavens D., Baker D., Clark M.D. (2019). A critical comparison of technologies for a plant genome sequencing project. Gigascience.

[196] Jung H., Jeon M.S., Hodgett M., Waterhouse P., Eyun S.I. (2020). Comparative Evaluation of Genome Assemblers from Long-Read Sequencing for Plants and Crops. J. Agricult. Food Chem..

[197] Li F.W., Harkess A. (2018). A guide to sequence your favorite plant genomes. Appl. Plant Sci..

[198] Hirsch C.N., Buell C.R. (2013). Tapping the Promise of Genomics in Species with Complex, Nonmodel Genomes. Annu. Rev. Plant Biol..

[199] McKain M.R., Johnson M.G., Uribe-Convers S., Eaton D., Yang Y. (2018). Practical considerations for plant phylogenomics. Appl. Plant Sci..

[200] Davey J.W., Hohenlohe P.A., Etter P.D., Boone J.Q., Catchen J.M., Blaxter M.L. (2011). Genome-wide genetic marker discovery and genotyping using next-generation sequencing. Nat. Rev. Genet..

[201] Pease J.B., Brown J.W., Walker J.F., Hinchliff C.E., Smith S.A. (2018). Quartet Sampling distinguishes lack of support from conflicting support in the green plant tree of life. Am. J. Bot..

[202] Bryant D., Moulton V. (2004). Neighbor-Net: An agglomerative method for the construction of phylogenetic networks. Mol. Biol. Evol..

[203] Pritchard J.K., Stephens M., Donnelly P. (2000). Inference of population structure using multilocus genotype data. Genetics.

[204] Falush D., Stephens M., Pritchard J.K. (2007). Inference of population structure using multilocus genotype data: Dominant markers and null alleles. Mol. Ecol. Notes.

[205] Frichot E., Mathieu F., Trouillon T., Bouchard G., Francois O. (2014). Fast and efficient estimation of individual ancestry coefficients. Genetics.

[206] Anderson E.C., Thompson E.A. (2002). A model-based method for identifying species hybrids using multilocus genetic data. Genetics.

[207] Blischak P.D., Chifman J., Wolfe A.D., Kubatko L.S. (2018). HyDe: A Python Package for Genome-Scale Hybridization Detection. Syst. Biol..

[208] Peralta M., Combes M.-C., Cenci A., Lashermes P., Dereeper A., Lashermes P. (2013). SNiPloid: A utility to exploit high-throughput SNP data derived from RNA-seq in allopolyploid species. Int. J. Pl. Genom..

[209] Eaton D.A.R., Hipp A.L., Gonzalez-Rodriguez A., Cavender-Bares J. (2015). Historical introgression among the American live oaks and the comparative nature of tests for introgression. Evolution.

[210] Malinsky M., Trucchi E., Lawson D.J., Falush D. (2018). RADpainter and fineRADstructure Population Inference from RADseq Data. Mol. Biol. Evol..

[211] Karbstein K., Tomasello S., Hodac L., Lorberg E., Daubert M., Hörandl E. (2021). Moving beyond assumptions: Polyploidy and environmental effects explain a geographical parthenogenesis scenario in European plants. Mol. Ecol..

[212] Hühn P., Dillenberger M.S., Gerschwitz-Eidt M., Hörandl E., Los J.A., Messerschmid T.F.E., Kadereit G. (2022). How challenging RADseq data turned out to favor coalescent-based species tree inference. A case study in *Aichryson* (Crassulaceae). Mol. Phy. Evol..

[213] Weitemier K., Straub S.C.K., Cronn R.C., Fishbein M., Schmickl R., McDonnell A., Liston A. (2014). Hyb-Seq: Combining target enrichment and genome skimming for plant phylogenomics. Appl. Plant Sci..

[214] Oxelman B., Brysting A.K., Jones G.R., Marcussen T., Oberprieler C., Pfeil B.E. (2017). Phylogenetics of Allopolyploids. Annu. Rev. Plant Biol..

[215] Rothfels C. (2021). Polyploid phylogenetics. New Phytol..

[216] Tiley G.P., Crowl A.A., Manos P.S., Sessa E.B., Solís-Lemus C., Yoder A.D., Burleigh J.G. (2021). Phasing alleles improves network inference with allopolyploids. bioRxiv.

[217] St Onge K.R., Foxe J.P., Li J.R., Li H.P., Holm K., Corcoran P., Wright S.I. (2012). Coalescent-based analysis distinguishes between allo- and autopolyploid origin in Shepherd’s Purse (*Capsella bursa-pastoris*). Mol. Biol. Evol..

[218] Brandrud M.K., Baar J., Lorenzo M.T., Athanasiadis A., Bateman R., Chase M.W., Paun O. (2020). Phylogenomic relationships of diploids and the origins of allotetraploids in *Dactylorhiza* (Orchidaceae). Syst. Biol..

[219] Pellino M., Hojsgaard D., Schmutzer T., Scholz U., Hörandl E., Vogel H., Sharbel T.F. (2013). Asexual genome evolution in the apomictic *Ranunculus auricomus* complex: Examining the effects of hybridization and mutation accumulation. Mol. Ecol..

[220] Lyu R., He J., Luo Y., Lin L., Yao M., Cheng J., Li L. (2021). Natural hybrid origin of the controversial “species” *Clematis × pinnata* (Ranunculaceae) based on multidisciplinary evidence. Front. Plant Sci..

[221] Turland N.J., Wiersema J.H., Barrie F.R., Greuter W., Hawksworth D.L., Herendeen P.S., Smith G.F. (2018). International Code of Nomenclature for Algae, Fungi, and Plants (Shenzhen Code) Adopted by the Nineteenth International Botanical Congress Shenzhen, China, July 2017.

[222] Ballere C.L., Trewavas A. (2009). Plant Behaviour Special Issue. Plant Cell Environ..

[223] Ulum F.B., Hadacek F., Hörandl E. (2021). Polyploidy improves photosynthesis regulation within the *Ranunculus auricomus* complex (Ranunculaceae). Biology.

[224] Coate J.E., Powell A.F., Owens T.G., Doyle J.J. (2013). Transgressive physiological and transcriptomic responses to light stress in allopolyploid *Glycine dolichocarpa* (Leguminosae). Heredity.

[225] Dong Y.T., Hu G.J., Yu J.W., Thu S.W., Grover C.E., Zhu S.J., Wendel J.F. (2020). Salt-tolerance diversity in diploid and polyploid cotton (*Gossypium*) species. Plant J..

